# Pre-natal clues of a genetic tale: how foetal heart rate foretells long QT syndrome

**DOI:** 10.1093/europace/euad322

**Published:** 2023-10-26

**Authors:** Babken Asatryan, Rebecca McClellan, Caridad M De La Uz

**Affiliations:** Division of Cardiology, Department of Medicine, Johns Hopkins University School of Medicine, 600 N Wolfe Str, Baltimore, MD 21287, USA; Division of Cardiology, Department of Medicine, Johns Hopkins University School of Medicine, 600 N Wolfe Str, Baltimore, MD 21287, USA; Division of Cardiology, Department of Medicine, Johns Hopkins University School of Medicine, 600 N Wolfe Str, Baltimore, MD 21287, USA; Division of Pediatric Cardiology, Department of Pediatrics, Pediatric and Congenital Cardiac Electrophysiology, Johns Hopkins University School of Medicine, Baltimore, MD, USA

**This editorial refers to ‘Effects of cohort, genotype, variant, and maternal β-blocker treatment on fetal heart rate predictors of inherited long QT syndrome’, by A.M. Kaizer *et al*. doi:10.1093/europace/euad319.**

In this nascent era of precision medicine, the unfolding tale of long QT syndrome (LQTS) shines as a luminous chapter and a prominent testament to what the future holds for genetic cardiovascular diseases.^1,2^ Initiating β-blocker therapy, possibly combined with mexiletine depending on genotype, has yielded remarkable outcomes in reducing morbidity and mortality in LQTS patients, and the imperative of early diagnosis and management in this context cannot be overstated.^3^ LQTS is a major cause of cardiac arrest in children and young adults^4,5^ and is causally implicated in 8–10% of sudden infant deaths^6,7^ and unexplained stillbirth.^8^ This understanding presents pre-natal diagnosis of LQTS as a pivotal window of opportunity to proactively mitigate the risk of life-threatening arrhythmias and sudden cardiac events in affected neonates and children.

Diagnosing LQTS in a foetus proves to be an arduous task as a reliable foetal ECG is not available, and magnetocardiography is only offered in a few specialized centres globally. While pre-implantation genetic testing or pre-natal genetic testing by way of chorionic villus sampling and amniocentesis can be performed to ascertain foetal LQTS genetic status, these procedures come with their own challenges and risks and many families elect to defer diagnosis to the post-natal period. In addition, these options are only available in babies where there is pre-existing knowledge of familial LQTS and a clear pathogenic variant.^9^ On the other hand, many—but not all^10^—experts believe that there is sufficient evidence to recommend routine ECG screening for LQTS in infancy.^11^ However, slight QT prolongation unrelated to LQTS might be seen in the early days of life, and there is a need for comprehensive assessment of ECG screening performance in this population.^10^

Another diagnostic avenue being explored is foetal heart rate (FHR), a biomarker widely used in multiple domains, that could raise suspicion for foetal LQTS. In early studies, FHR of <110 bpm at any gestational age (GA)—the obstetric definition of foetal bradycardia—was employed as an indicator warranting consideration of LQTS. It was later demonstrated in a Swedish LQT1 founder cohort that FHR ≤ 3rd percentile for GA offers a better performance, boasting a specificity of 97% while exhibiting sensitivity of 50% for detecting foetal LQTS,^12^ underscoring the need for better FHR-based prediction models.

In this issue of the journal, the Fetal LQTS Consortium presents an elegant study that informs the natural history of LQTS by expanding and refining the current understanding of FHR/GA dynamic in familial LQTS pregnancies, taking cohort, genotype, variant type/effect, and maternal β-blocker therapy into consideration.^13^ The authors used a multi-source identification to recruit an extensive cohort of 267 offspring from 164 LQTS families across eight countries, of which 60% inherited the familial LQTS-causative variant [LQT1 (*KCNQ1*, 82%), LQT2 (*KCNH2*, 13%), or LQT3 (*SCN5A*, 5%)]. Since some of the earlier data regarding the FHR/GA relationship relied on two Swedish LQT1 Founder populations,^12^ the authors included both the Swedish Founder LQT1 cases and other inherited LQT1 foetuses to assess potential differences. Their analysis, utilizing a linear mixed effects regression model, controlled for GA, maternal β-blocker use, and the interaction between β-blocker use and foetal LQT1 status, revealed no significant differences in the mean FHRs between the founder cohort and other LQT1 foetuses. Taking precision to the next level, the authors unveiled for the first time that in LQT1 foetuses, FHR is affected by both the *KCNQ1* variant type and its functional impact on current density and β-adrenergic response, with lowest FHRs observed in foetuses hosting non-nonsense variants and variants leading to severe functional loss (i.e. current density ≤ 25% of wild type or impaired β-adrenergic response). Exposure to β-blockers *in utero* potentiated the intrinsically low FHRs in foetuses with LQTS without notable effect on those without LQTS, further supporting the safety profile of β-blocker treatment.

Leveraging this extensive dataset, existing prediction schemes for foetal LQTS were put to the test. The obstetric definition of foetal bradycardia exhibited an accuracy of only 45%, failing to identify 92% of LQT1 and LQT2 cases. As expected, employing the previously proposed definition of foetal bradycardia as FHR < 3rd percentile for GA showed improved accuracy at 74%, with a decent sensitivity of 84.9%, albeit with an unacceptably low specificity of 59.2%. This contrasts with the performance of this approach in the Swedish Founder LQT1 cohort, exhibiting an inversely lower sensitivity but higher specificity,^12^ indicating the low reproducibility of this scheme. Finally, the authors went on develop a novel prediction model using GA as a continuous predictor to estimate the probability that a foetus is LQTS genotype-positive from a logistic regression model, assuming 50% prevalence. Compared to the FHR < 3rd percentile for GA model, this strategy demonstrated similar accuracy with a better balance between sensitivity (71.1%) and specificity (80.6%) for identifying LQT1 and LQT2 foetuses.

The authors of this study should be commended for compiling such a large and rare cohort of unique cases, delivering more insights into the intricate links between foetal bradycardia and congenital LQTS, and offering an improved screening model for prediction of foetal LQT1 and LQT2. A few limitations would benefit from further research and validation. While the investigation shed light on the effects of β-blocker therapy on FHR, a deeper exploration of maternal β-blocker therapy would benefit from investigating dose–response relationships, β-blocker type, treatment duration, and the impact of timing and compliance on FHR in the context of different LQTS genotypes. Interestingly, only 46% (83/179) of LQTS mothers in the study were treated with β-blockers during pregnancy, mostly due to five-fold lower treatment rates in the Swedish Founder cohort (16%) as compared to LQT1 and LQT2 cases from international centres (83%); this is explained by the historical nature of the Swedish Founder cohort, recruited in 1980s. This indicates the need for physician reassurance on β-blocker safety (and importance) during pregnancy in women with LQTS to increase treatment rates and improve outcomes. Additionally, while promising, the novel LQTS prediction model requires rigorous external validation and cross-validation to assess its applicability beyond the study cohort and ensure its reliability in real-world clinical practice for perinatal LQTS prediction. Additionally, the isolated detection of FHR using Doppler or cardiotocography, without evaluation of atrioventricular relationship with foetal echo/m-mode or magnetocardiograpy, may yield false positive concerns for foetal LQTS in the general population where other causes of foetal bradycardia are much more prevalent. Numerous other conditions have been associated with primary and secondary foetal bradycardia including complete and high-grade atrioventricular (AV) block caused by maternal autoantibodies, structural heart disease, or unknown aetiologies, foetal distress, non-LQTS monogenetic disorders (e.g. *PRKAG2*-associated glycogen storage disorders, Holt–Oram syndrome, short QT syndrome, tuberous sclerosis, and sick sinus syndrome associated with *HCN4* or *SCN5A* mutations), and other systemic diseases.^14^ There are limited data on the expected heart rates of foetuses with the aforementioned bradycardic conditions, and this may significantly impact the specificity of this model in an unselected population. Discerning the specific type of bradyarrhythmia, which is critical to ruling out other more common causes of foetal bradycardia, would require evaluation of heart rate and AV relationship using more costly modalities to limit false positive screenings for LQTS. This, in turn, may limit the cost-effectiveness and the accessibility of such a screening. Further investigation can also help identify unique characteristics of foetuses with missed LQTS diagnoses using this model, thereby informing further refinements. For instance, it would be interesting to see whether missed cases could be effectively identified through pulsed Doppler-based measurements of left ventricular isovolumetric relaxation—another marker with diagnostic potential.^15^ Lastly, data remain limited on prediction of foetal LQT3 and *de novo* LQTS (for all genetic subtypes), which tend to present with frequent and lethal post-natal cardiac arrhythmias.^12,16^ Conducting longitudinal screening and implementation studies in large populations, combining the proposed FHR/GA threshold model with pre-/post-natal genetic testing in foetuses with complex and novel rhythms, can be pivotal in advancing diagnostic protocols to a higher level of accuracy for both inherited and *de novo* forms of LQTS.

## Data Availability

No original data was used for this article.

## Notes

**Funding:** B.A. is supported by a post-doctoral research fellowship grant from the Gottfried und Julia Bangerter-Rhyner-Stiftung (Switzerland) and the 2022 Research Fellowship for aspiring electrophysiologists from the Swiss Heart Rhythm Foundation.
